# Supplementary material for: Improving the success of reinforcement programs: effects of a two-week confinement in a field enclosure on the anti-predator behaviour of captive-bred European hamsters
Source: PeerJ. 2023 Sep 1;11:e15812. doi: 10.7717/peerj.15812 (PMC10476607; doi:10.7717/peerj.15812)
Supplement: Supplemental Information 1 [file peerj-11-15812-s001.docx]

| **Observed behaviour** | **Definition** | **Variables included in data analysis** |
| --- | --- | --- |
| Attack_Bite | bites the fox | **Number of attacks on fox model** |
| Attack_Jump | jumps in front of or onto fox |  |
| Latency before hiding | time elapsed before entry into APT at start of phase 2 | **Latency before first entry into APT** |
| Enter.tube | all four limbs inside APT | **Time (%) inside APT** |
| Exit.tube | all four limbs outside APT |  |
| Inspect | smelling arena and/or tube | **Time (%) exploring arena when outside APT** |
| Walk_slow/exploration | moves around slowly, smelling while walking |  |
