# Supplementary material for: Improving the success of reinforcement programs: effects of a two-week confinement in a field enclosure on the anti-predator behaviour of captive-bred European hamsters
Source: PeerJ. 2023 Sep 1;11:e15812. doi: 10.7717/peerj.15812 (PMC10476607; doi:10.7717/peerj.15812)
Supplement: Supplemental Information 2 [file peerj-11-15812-s002.docx]

| Response variable | Model distribution used | Model package (R) | Post-hoc |
| --- | --- | --- | --- |
| (i) time (%) inside APT | Quasi-binomial (overdispersion) | MASS (glmmPQL) | Tuckey |
| (ii) time (%) exploring the arena when outside APT |  |  |  |
| (iii) latency between fox model presentation and shelter (APT) seeking | Gamma |  |  |
| (iv) number of hamster attacks on fox model | Quasi-poisson (count data and overdispersion) |  |  |
