# Supplementary material for: Improving the success of reinforcement programs: effects of a two-week confinement in a field enclosure on the anti-predator behaviour of captive-bred European hamsters
Source: PeerJ. 2023 Sep 1;11:e15812. doi: 10.7717/peerj.15812 (PMC10476607; doi:10.7717/peerj.15812)
Supplement: Supplemental Information 3 — Significant differences are indicated by asterisks. Some behavioural variables are specific to a particular trial phase. [file peerj-11-15812-s003.docx]

|  |  | Phase 1 (before confrontation) | | | | Phase 2 (during confrontation) | | | | Phase 3 (after confrontation) | | | |
| --- | --- | --- | --- | --- | --- | --- | --- | --- | --- | --- | --- | --- | --- |
|  | Source | Estimate ± SE | t-value | Df | P-value | Estimate ± SE | t-value | Df | P-value | Estimate ± SE | t-value | Df | P-value |
| Time (%) spent inside APT | (Intercept) | **-2.44 ± 0.33** | **-7.33** | **20** | **<0.001***** | 1.59 ± 0.53 | 3.01 | 20 | <0.01** | -0.52 ± 0.42 | -1.24 | 20 | 0.23 |
|  | Group | 1.12 ± 0.43 | 2.63 | 20 | 0.46 | 0.74 ± 0.84 | 0.87 | 20 | 0.12 | 1.71 ± 0.66 | 2.61 | 20 | 0.84 |
|  | Test | 0.91 ± 0.36 | 2.49 | 20 | 0.88 | 1.04 ± 0.55 | 1.88 | 20 | 0.14 | 0.82 ± 0.50 | 1.66 | 20 | 0.27 |
|  | Group*Test | **-1.74 ± 0.53** | **-3.28** | **20** | **<0.01**** | **-3.47 ± 0.81** | **-4.28** | **20** | **<0.001***** | **-3.15 ± 0.79** | **-3.97** | **20** | **<0.001***** |
| Time (%) exploring when outside APT | (Intercept) | -0.48 ± 0,20 | -2.40 | 20 | 0.03 |  | | | | -1.68 ± 0.35 | -4.84 | 20 | 0.0001*** |
|  | Group | -0.28 ± 0,30 | -0.92 | 20 | 0.62 |  |  |  |  | -1.32 ± 0.69 | -1.98 | 20 | 0.61 |
|  | Test | **0.09 ± 0,23** | **0.39** | **20** | **<0.01**** |  |  |  |  | 0.006 ± 0.4 | 0.01 | 20 | 0.11 |
|  | Group*Test | **0.75 ± 0,34** | **2.19** | **20** | **<0.05*** |  |  |  |  | **1.61 ± 0.72** | **2.22** | **20** | **<0.05*** |
| Latency before first entry into APT | (Intercept) |  | | | | <0.001 ± 0.001 | 0.72 | 20 | 0.48 |  | | | |
|  | Group |  |  |  |  | 0.08 ± 0.04 | 1.75 | 20 | 0.59 |  |  |  |  |
|  | Test |  |  |  |  | 0.04 ± 0.03 | 1.64 | 20 | 0.61 |  |  |  |  |
|  | Group*Test |  |  |  |  | **-0.45 ± 0.15** | **-2.96** | **20** | **<0.01*** |  |  |  |  |
| Nr. of hamster attacks | (Intercept) |  | | | | -1.01 ± 0.84 | -1.20 | 20 | 0.25 |  | | | |
|  | Group |  |  |  |  | 0.86 ± 1.14 | 0.76 | 20 | 0.46 |  |  |  |  |
|  | Test |  |  |  |  | -0.83 ± 0.57 | -1.45 | 20 | 0.16 |  |  |  |  |
|  | Group*Test |  |  |  |  | **1.73 ± 0.61** | **2.79** | **20** | **<0.05*** |  |  |  |  |
