# Supplementary material for: Improving the success of reinforcement programs: effects of a two-week confinement in a field enclosure on the anti-predator behaviour of captive-bred European hamsters
Source: PeerJ. 2023 Sep 1;11:e15812. doi: 10.7717/peerj.15812 (PMC10476607; doi:10.7717/peerj.15812)
Supplement: Supplemental Information 4 — Test #1: initial test; test #2: after treatment; test #3: 1 month after treatment. Significant differences are indicated by asterisks. Some behavioural variables are specific to a particular test phase. [file peerj-11-15812-s004.docx]

|  |  | Phase 1 (before confrontation) | | | | Phase 2 (during confrontation) | | | | Phase 3 (after confrontation) | | | |
| --- | --- | --- | --- | --- | --- | --- | --- | --- | --- | --- | --- | --- | --- |
|  |  | Estimate ± SE | t-value | Df | P-value | Estimate ± SE | t-value | Df | P-value | Estimate ± SE | t-value | Df | P-value |
| Time (%) spent inside APT | (Intercept) | **-2.15 ± 0.44** | **-4.89** | **17** | **<0.001***** | -0.03 ± 0.52 | -0.06 | 17 | 0.95 | -1.11 ± 0.56 | -1.98 | 17 | 0.064 |
|  | Test2 | 0.83 ± 0.52 | 1.59 | 17 | 0.13 | **2.09 ± 0.59** | **3.53** | **17** | **<0.01**** | **2.27 ± 0.74** | **3.07** | **17** | **<0.01**** |
|  | Test 3 | 0.61 ± 0.55 | 1.12 | 17 | 0.28 | **1.82 ± 0.59** | **3.10** | **17** | **<0.01**** | 1.37 ± 0.71 | 1.93 | 17 | 0.07 |
| Time (%) exploring when outside APT | (Intercept) | **-5.25 ± 0.13** | **-40.68** | **17** | **<0.001***** |  | | | | **-6.19 ± 0.28** | **-21.74** | **17** | **<0.001***** |
|  | Test2 | **-0.49 ± 0.21** | **-2.35** | **17** | **<0.05*** |  |  |  |  | **-1.41 ± 0.59** | **-2.39** | **17** | **<0.05*** |
|  | Test 3 | **-0.67 ± 0.23** | **-2.92** | **17** | **<0.01**** |  |  |  |  | -0.65 ± 0.46 | -1.41 | 17 | 0.18 |
| Latency before first entry into APT | (Intercept) |  | | | | **0.01 ± 0.01** | **2.25** | **17** | **<0.05*** |  | | | |
|  | Test2 |  |  |  |  | **0.27 ± 0.12** | **2.15** | **17** | **<0.05*** |  |  |  |  |
|  | Test 3 |  |  |  |  | 0.04 ± 0.02 | 1.59 | 17 | 0.13 |  |  |  |  |
| Nr. of hamster attacks | (Intercept) |  | | | | **-0.78 ± 0.29** | **-2.71** | **17** | **<0.01**** |  | | | |
|  | Test2 |  |  |  |  | **2.17 ± 0.09** | **22.82** | **17** | **<0.001***** |  |  |  |  |
|  | Test 3 |  |  |  |  | **1.97 ± 0.19** | **10.57** | **17** | **<0.01**** |  |  |  |  |
