# Supplementary material for: Improving the success of reinforcement programs: effects of a two-week confinement in a field enclosure on the anti-predator behaviour of captive-bred European hamsters
Source: PeerJ. 2023 Sep 1;11:e15812. doi: 10.7717/peerj.15812 (PMC10476607; doi:10.7717/peerj.15812)
Supplement: Supplemental Information 5 — When under attack (phase 2), hamsters never explored the arena, as indicated by NA. [file peerj-11-15812-s005.docx]

|  | **Variables** | **Phase** | **Estimate± SE** | ***Z*** | ***Df*** | **p** |
| --- | --- | --- | --- | --- | --- | --- |
| Test #1: Control group vs. Field group | Time spent in the APT | 1 | -0.61±0.40 | -1.53 | 20 | 0.41 |
|  |  | 2 | -2.74±0.79 | -3.47 | 20 | 0.003* |
|  |  | 3 | -1.44±0.61 | -2.34 | 20 | 0.09 |
|  | Exploration | 1 | 0.48±0.28 | 1.70 | 20 | 0.32 |
|  |  | 2 | NA | NA | NA | NA |
|  |  | 3 | 0.29±0.48 | 0.60 | 20 | 0.93 |
|  | Latency before first enter the APT | 2 | -0.20±0.08 | -2.42 | 20 | 0.06 |
|  | Attack on the fox model | 2 | 1.73±0.62 | 2.79 | 20 | 0.08 |
