# Supplementary figures and images for: Improving the success of reinforcement programs: effects of a two-week confinement in a field enclosure on the anti-predator behaviour of captive-bred European hamsters
Source: PeerJ. 2023 Sep 1;11:e15812. doi: 10.7717/peerj.15812 (PMC10476607; doi:10.7717/peerj.15812)

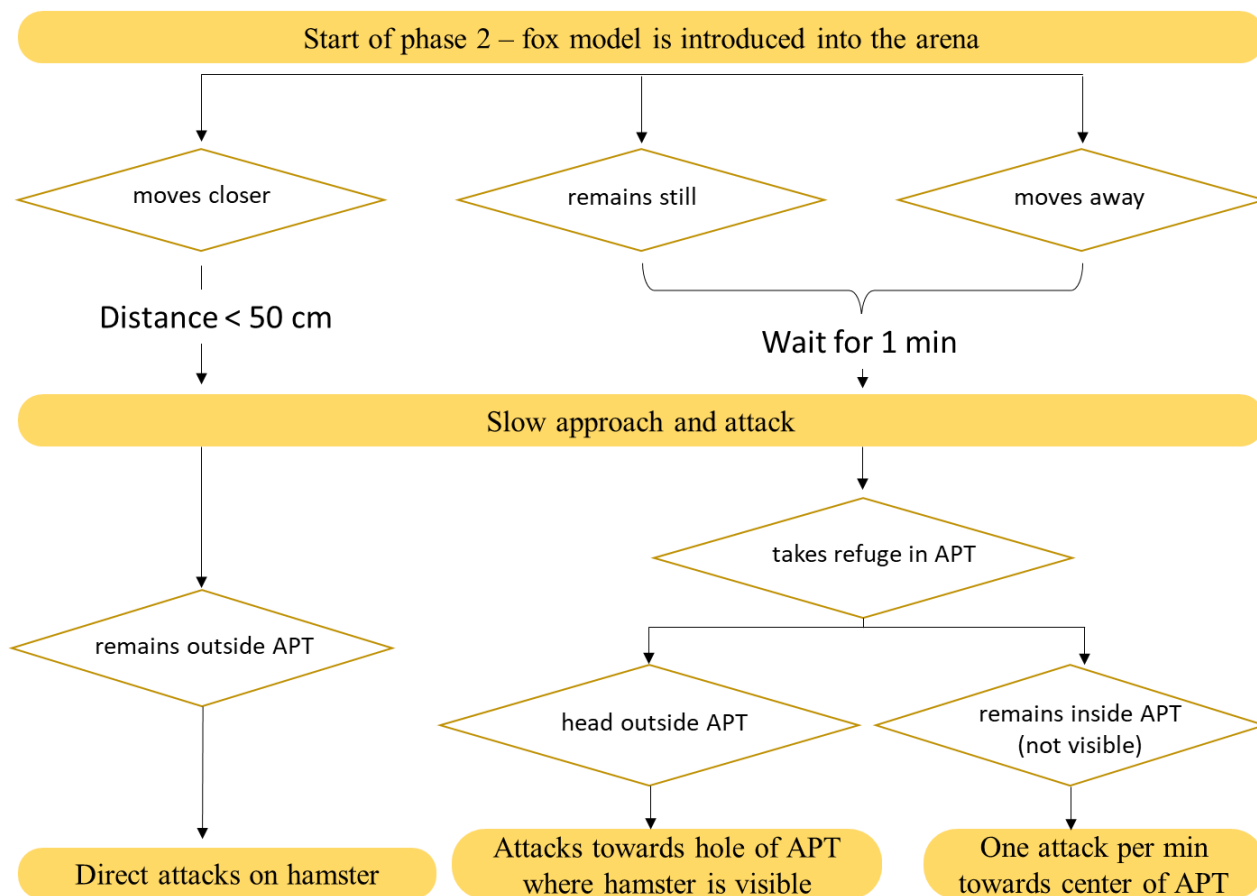

Supplement: Supplemental Information 6 — The diamond-shaped boxes indicate the behaviour of the hamster, while reactions of the fox model, directed by the experimenter according to hamster behaviour, are shown inside the yellow boxes. [file peerj-11-15812-s006.pdf]

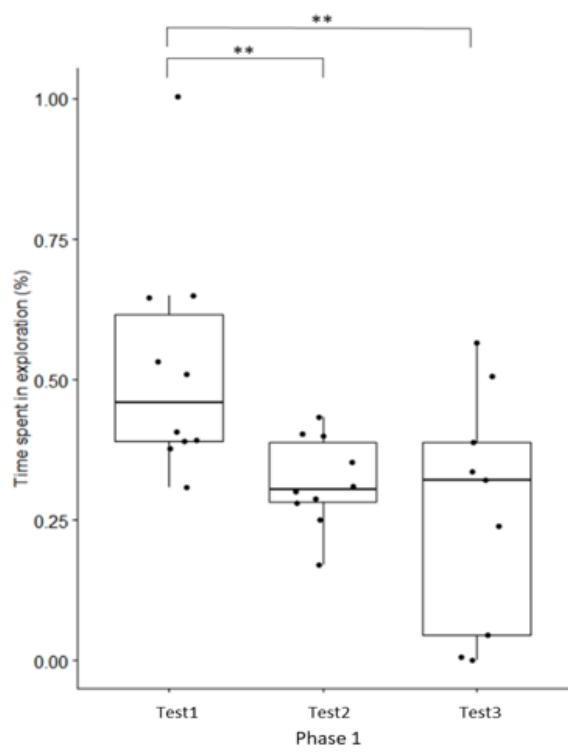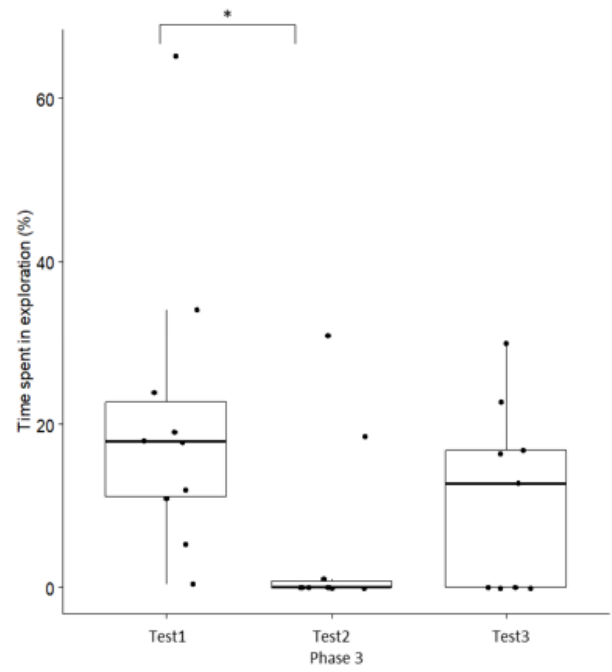

Supplement: Supplemental Information 7 — Individuals are indicated by black dots and significant differences between tests are indicated by asterisks. [file peerj-11-15812-s007.pdf]

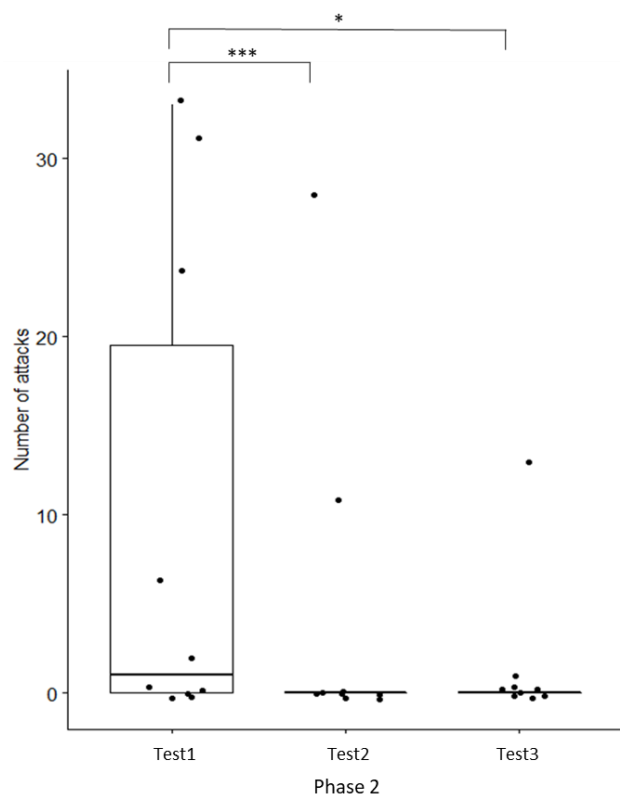

Supplement: Supplemental Information 8 — Individuals are indicated by black dots and significant differences between tests are indicated by asterisks. [file peerj-11-15812-s008.pdf]
